# Isoform Diversity and Regulation in Peripheral and Central Neurons Revealed through RNA-Seq

**DOI:** 10.1371/journal.pone.0030417

**Published:** 2012-01-17

**Authors:** Jessica K. Lerch, Frank Kuo, Dario Motti, Richard Morris, John L. Bixby, Vance P. Lemmon

**Affiliations:** 1 The Miami Project to Cure Paralysis, Miller School of Medicine, University of Miami, Miami, Florida, United States of America; 2 The Hussman Institute for Human Genomics, Miller School of Medicine, University of Miami, Miami, Florida, United States of America; 3 Department of Cellular and Molecular Pharmacology, Miller School of Medicine, University of Miami, Miami, Florida, United States of America; 4 Department of Neurological Surgery, Miller School of Medicine, University of Miami, Miami, Florida, United States of America; Indiana University School of Medicine, United States of America

## Abstract

To fully understand cell type identity and function in the nervous system there is a need to understand neuronal gene expression at the level of isoform diversity. Here we applied Next Generation Sequencing of the transcriptome (RNA-Seq) to purified sensory neurons and cerebellar granular neurons (CGNs) grown on an axonal growth permissive substrate. The goal of the analysis was to uncover neuronal type specific isoforms as a prelude to understanding patterns of gene expression underlying their intrinsic growth abilities. Global gene expression patterns were comparable to those found for other cell types, in that a vast majority of genes were expressed at low abundance. Nearly 18% of gene loci produced more than one transcript. More than 8000 isoforms were differentially expressed, either to different degrees in different neuronal types or uniquely expressed in one or the other. Sensory neurons expressed a larger number of genes and gene isoforms than did CGNs. To begin to understand the mechanisms responsible for the differential gene/isoform expression we identified transcription factor binding sites present specifically in the upstream genomic sequences of differentially expressed isoforms, and analyzed the 3′ untranslated regions (3′ UTRs) for microRNA (miRNA) target sites. Our analysis defines isoform diversity for two neuronal types with diverse axon growth capabilities and begins to elucidate the complex transcriptional landscape in two neuronal populations.

## Introduction

Next Generation sequencing applied to the transcriptome (RNA-Seq) is a transformational technology that uncovers vast amounts of novel information about transcript expression and identity. With its advent the rich complexity of the mammalian transcriptome is beginning to be fully appreciated [1]–[3]. The most common ways of studying gene expression and function (knockout, knockdown, transgenic overexpression, and microarray) have generally been unable to distinguish between different isoforms from a given locus, or even to reveal their number and complexity. For example, gene knockout or knockdown may (or may not) affect all isoforms, and overexpression experiments are generally limited to a single “standard” isoform. Isoforms of the same gene can differ in expression pattern and function. For example, alternative splicing of the chromatin remodeling factors Brg1-associated factors (BAF57) produces two different isoforms, one that is glial specific and one that is neuronal specific [4]. Neural cell adhesion molecule (NCAM) has three known isoforms and loss of the 180 kDa version leads to changes at the neuromuscular junction that are accompanied by motor deficits [5]. In addition to isoforms that differ in protein coding region, changes in the UTRs can effect isoform localization. This is illustrated by a brain derived neurotrophic factor (BDNF) isoform that has a long 3′ UTR which is necessary for localization to dendrites [6]. Systematic characterization of the identity and number of isoforms, or the diversity of transcripts expressed from any single transcriptional unit has not been possible without laborious experimentation, so the structural and functional diversity of transcripts from the vast majority of genes is not understood. RNA-Seq has the ability to uncover details about isoform diversity and expression levels. It offers a large dynamic range, accurate quantification and the ability to identify the sequences of all the RNA species within a given cell type. This information enables a comprehensive approach to identification of the molecular networks and regulatory mechanisms underlying transcriptional control.

A detailed knowledge of the expression of cell specific isoforms is crucial to understanding cellular diversity. This is especially obvious in the nervous system, with its enormous variety of cell types with distinct functions and characteristics. Previous RNA-Seq studies of nervous system tissues have involved mixed populations of neurons with other cell types [7], [8]. To identify neuronally expressed isoforms, and to relate gene expression to neuronal type-specific properties, we applied RNA-Seq to cultured peripheral neurons from dorsal root ganglia (DRG neurons) and to cultured cerebellar granule neurons (CGNs). This neuronal comparison should lend itself to the identification of isoforms and pathways pertinent to the intrinsic mechanisms underlying axon regeneration, since DRG neurons regenerate in situations in which central nervous system neurons, such as CGNs, do not [9], [10].

We found enormous diversity of isoform expression between DRG neurons and CGNs, with over 8,000 differentially expressed isoforms. We scanned the promoters and 3′ UTRs of differentially expressed isoforms for cis-elements involved in transcription regulation and identified transcription factors (TFs) and miRNAs potentially involved in the control of isoform specific expression. Known interactions between predicted TFs and miRNAs were used to generate regulatory networks that may be driving isoform specific expression differences. Thus we have identified new intracellular targets that may affect neuronal type specific transcript expression.

## Results

### Next Generation Transcriptome Sequencing

RNA-Seq was performed on the polyadenylated fraction of RNA isolated from DRG neurons and CGNs from postnatal day 8 mice grown on the growth permissive substrate laminin (LN). DRG neurons were used because of their robust axonal growth in culture, and because they represent a well-established model for understanding axonal regeneration [9]–[11]. CGNs were chosen because they can be obtained in relatively large numbers and high purity and have been used extensively for in vitro studies of neurite growth [12], [13]. Approximately 40 million, 50 base pair sequence fragments (“reads”) were recovered from each biological replicate (Table 1). Read alignment, transcript assembly and expression estimation were performed using Bowtie, Tophat and Cufflinks software [14]–[16]. ∼80% of all reads mapped to the mouse reference genome (NCBI37/mm9) and over 3 million reads were recognized as spanning a splice junction (Table 1). Estimated normalized expression levels were reported in **F**ragments (aka: reads) **P**er **K**ilobase of exon per **M**illion mapped reads (FPKM). There were over 50,000 transcripts mapping to annotated areas of the genome and over 135,000 transcripts mapping to genomic regions lacking annotation. For the purpose of this report we focused only on transcription events at the level of known, active loci (annotated loci, Table S1, GEO Accession #GSE33343).

**Table 1:** Summary of the read alignment and mapping from Tophat.

Sample	Reads Processed	Reads with at least one reported alignment	Spliced Fragments	Reads Failing Alignment	Total Alignments
DRG1	39,925,227	32,659,840 (81.80%)	3,512,810	6,903,205 (17.29%)	59,973,578
DRG2	36,127,068	29,835,103 (82.58%)	3,752,635	5,928,924 (16.41%)	49,178,992
DRG3	37,869,918	30,129,189 (79.56%)	4,296,280	7,358,428 (19.43%)	48,293,420
CGN1	41,163,218	33,827,887 (82.18%)	4,678,223	6,813,356 (16.55%)	55,644,984
CGN2	44,523,431	36,476,724 (81.93%)	4,294,769	7,590,331 (17.05%)	62,193,644

**Figure 1:**
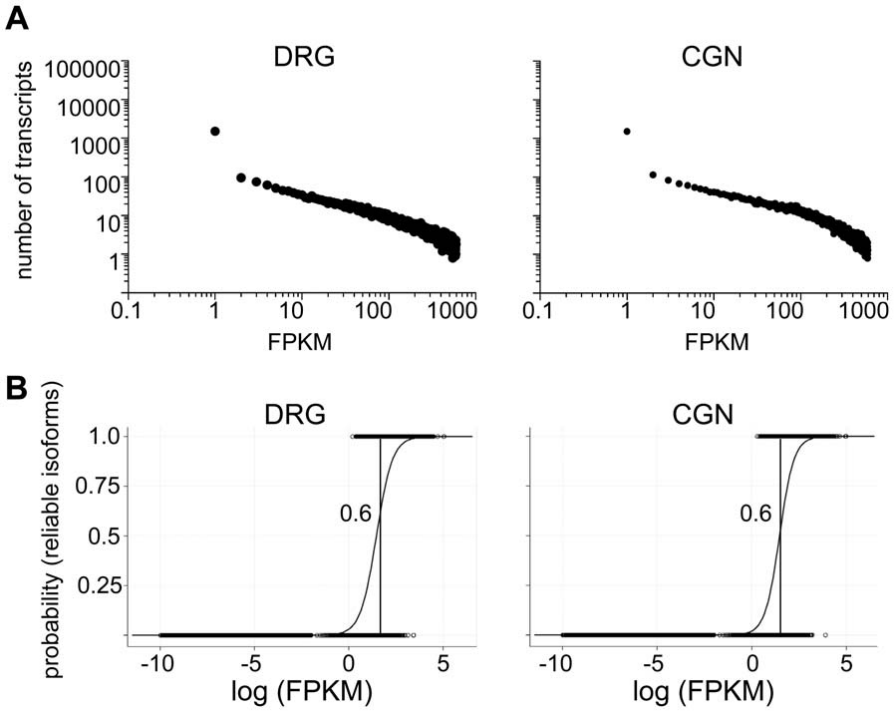
Patterns of transcript expression and determination of reliably expressed transcripts. *A*, Transcripts that occur at low abundances are more frequent than transcripts that occur at high abundances. Power-law distribution states that the probability of gene expression k, will decay as a power-law P(k) ∞ k^−r^. This expression pattern occurs in both neuronal types. DRG R^2^ = 0.927. CGN R^2^ = 0.902. Axis in log scale. *B*, Logistic regression relationships between ‘reliability’ of isoform expression, measured by isoform FPKM having a lower confidence limit exceeding 0, and log (FPKM), for DRG neurons (left) and CGNs (right).

The vast majority of transcripts mapping to an annotated locus (95%) had an FPKM that was lower than 1% of the FPKM of the highest expressed isoform (Tubb3, FPKM  = ∼400,000+) indicating that the majority of transcripts fall into a relatively low expression fraction. This observation is consistent with previously described global gene expression patterns (Figure 1A; [17]). To interpret patterns of transcript diversity within the limits of sequencing depth achieved in this study (40 million reads/sample), we identified a statistical cutoff for reliability of expression measurements based on FPKM values. The Cufflinks software produces estimates of FPKM and their 95% confidence intervals; we classified isoforms with a lower confidence bound >0 as having a “reliable” FPKM estimate and isoforms with a lower confidence bound equal to 0 as having an “unreliable” FPKM estimate. We quantified the relationship between reliability and FPKM using data from each group of biological replicates to fit a logistic regression function relating the probability that an isoform is reliable to its FPKM value (Figure 1B). Based on these analyses, we chose an FPKM threshold of 50. This threshold for FPKM produced nearly balanced false positive and false negative classification rates (false positive rates, ca. 0.14; false negative rates ca. 0.19). Unreliable transcript abundance values were considered to be not expressed for the purposes of our data analysis. In our data set there were 36,119 distinct transcripts with FPKM values ≥50 that mapped to annotated regions of the genome.

### Isoform Diversity

**Figure 2:**
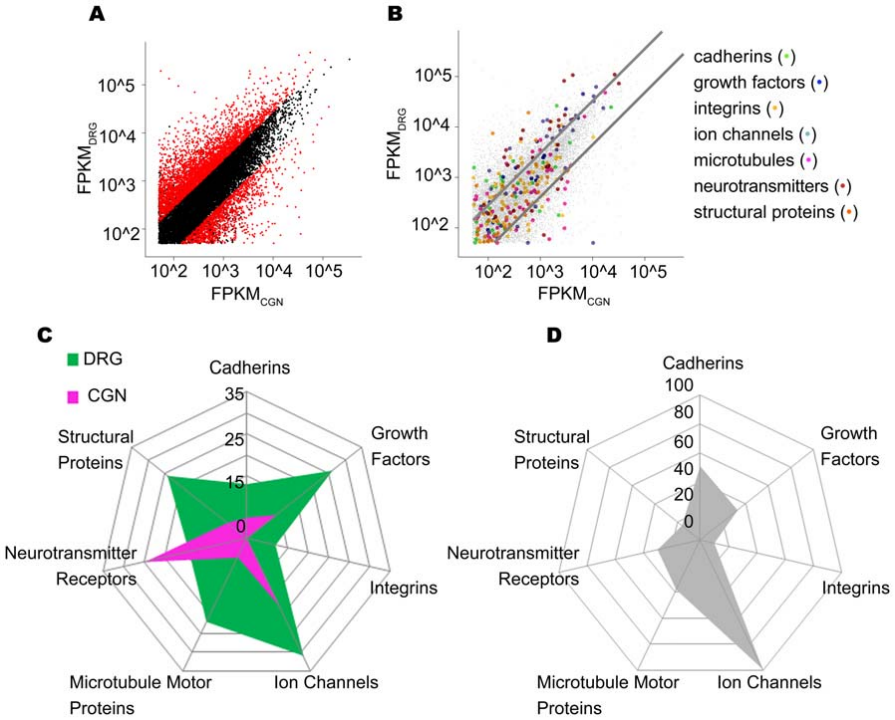
Differential isoform expression between DRGs and CGNs. *A*, Normalized abundances (FPKM) of isoforms in DRG neurons plotted against abundances in CGNs. Differentially expressed isoforms are in red. The criteria for differential expression are: | ln(FPKM_CGN_ / FPKM_DRG_) | >1; p<0.05, the statistical test was deemed acceptable by Cuffdiff and FPKM >50. *B*, Same plot as in *A* except differentially expressed isoforms falling within gene families of particular importance in neurons are indicated by different colored dots. The lines mark the efold changes of +1 and -1. *C,* The number of differentially expressed isoforms is represented for each group of genes. DRG neuron isoforms are in green and CGN isoforms are in purple. *D,* The number of isoforms found in common in DRG neurons and CGNs in each category. See Table 2.

Because DRG neurons and CNS neurons such as CGNs are highly distinct in their developmental origins, integration into circuits, and axonal growth properties [18]–[20], one would predict functionally important differences in their expression of genes and gene isoforms. We found that, while 10,365 genes were expressed by both populations of neurons, 5,328 genes were uniquely expressed by DRG neurons, and 4,358 genes were uniquely expressed by CGNs. One strength of RNA-Seq is the ability to identify differential patterns of isoform expression [2]. To address this issue, we defined isoforms as transcripts from the same gene that differ in their transcription start site (TSS), coding DNA sequence (CDS), and/or in the 3′ untranslated region (3′UTR). The majority of annotated loci produced isoforms found in both neuronal types (over 19,000; Table S1). Of the 19,000 shared isoforms, over 4600 were differentially expressed (Cuffdiff; see Materials and Methods); of these the majority were expressed significantly higher in DRG neurons compared to CGNs (Figure 2A; 3397 overexpressed in DRGs versus 1204 overexpressed in CGNs).

DRG neurons not only expressed higher levels of transcripts but also expressed approximately 25% more unique isoforms (transcripts expressed in one neuronal type but not the other). The 5328 cell type specific genes in DRG neurons produced 8483 isoforms, while CGNs had 4358 cell type specific genes that produced 6778 isoforms. Interestingly, the isoforms produced by DRG neurons were much more diverse, as a group, than those produced by CGNs. For example, isoforms expressed by DRG neurons utilized 1999 different transcription start sites (TSSs) compared to 710 used by CGN exclusive genes. Similarly, DRG neurons not only use a greater number of coding sequences (CDSs; 2050 vs. 747) but also a greater number of 3′ UTRs (1828 vs. 676), compared to CGNs. Overall, DRG neurons exhibited a nearly 3-fold increase in the number of TSS, CDS, and alternative 3′UTRs compared to transcripts found in CGNs. Thus DRG neurons not only express more isoforms, but each isoform differs, on average, in more positions than in CGNs.

To begin to understand the nature of the gene expression differences between these two neuronal types, we chose 7 gene classes relevant to neuronal functions such as cell adhesion, vesicle transport and neurotransmitter expression (cadherins; integrins; growth factors; ion channels; microtubule motor proteins; neurotransmitter receptors; and structural proteins). CGNs expressed a greater number of neurotransmitter receptor genes. In all other categories DRG neurons expressed the largest number of isoforms (Figure 2B, C; Table 2). Overall our data suggest that DRG neurons have a larger transcriptional repertoire compared to CGNs.

**Table 2:** Categories of differentially expressed isoforms.

Category	DRG	CGN	BOTH
Cadherins	13	5	51
Growth Factors	26	9	33
Integrins	7	0	10
Ion Channels	31	18	99
Microtubule Motor Proteins	22	5	38
Neurotransmitter Receptors	14	25	30
Structural Proteins	24	6	16

The number of isoforms overexpressed in DRGs or in CGNs is shown in each column. The number in the BOTH column reflects the number of isoforms which are expressed in both cell types (FPKM>50).

### Isoform Variation in Regeneration-Related Genes

**Figure 3:**
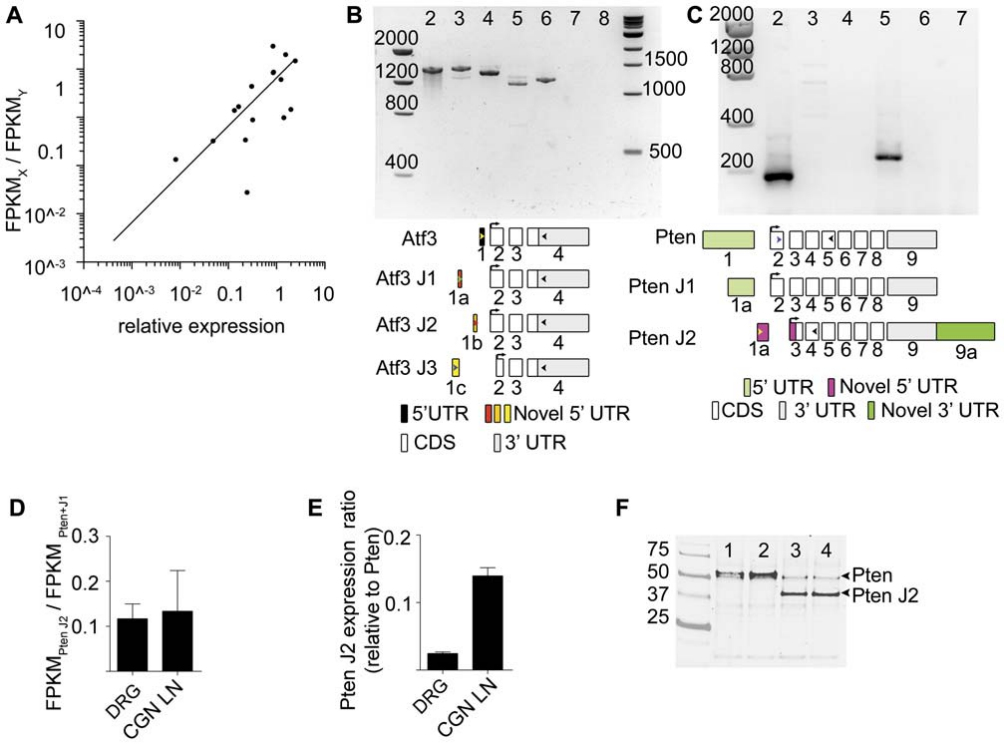
Validation and functional testing of novel isoforms. *A*, Expression of 15 isoforms was assessed by qPCR in DRG neurons and CGNs. Isoform expression was compared to the fold change in FPKM. (Relative expression and FPKM ratios in Table 3). *B*, *C*, *Atf3* and *Pten* isoform specific primers (bottom panels) were used on DRG cDNA and produced PCR products of the predicted sizes. *B*, Lane2: *Atf3* cDNA (Open BioSystems); Lane3: conventional *Atf3*; Lane4: *Atf3 J1*; Lane5: *Atf2 J2*; Lane 6: *Atf3 J3*; Lane7: no template control; Lane 8: no reverse transcription (RT) control. *C*, Lane2: conventional Pten; Lane3: no template control for *Pten*; Lane4: no RT control for *Pten*; Lane5: *Pten J2*; Lane 6: no template control for *Pten J2*; Lane 7: no RT control for *Pten J2*. Schematic representations of *Atf3* and *Pten* isoforms are below each gel (not to scale). Primer positions are indicated with colored arrows. *D*, The ratio of *Pten J2* expression to *Pten* conventional + *Pten J1* expression (in FPKM). The FPKMs for *Pten* and *Pten J1* were summed because there is no way to distinguish the isoforms by PCR. *Pten J2* expression is reduced 80–90%. *E*, qPCR validates the reduction in *Pten J2* expression. *F*, Western blot for PTEN (50 kD) and PTEN J2 (32 kD) confirms that both proteins can be produced from the corresponding cDNAs.

To evaluate the reproducibility of our results, we used qPCR to assess gene expression from 9 genes and compared these levels to those estimated by RNA-Seq (Tables 3 and 4). We chose amyloid beta (A4) precursor-like protein 1 (*Aplp1*) and ATPase, Na+/K+ transporting, alpha 3 polypeptide (*Atp1a3*) because the expression of these isoforms was estimated to be similar between the cell types; activating transcription factor 3 (*Atf3*) and the phosphatase and tensin homologue (*Pten*) were chosen because they gave rise to a host of unique isoforms; tissue inhibitor of metalloproteinase 1 (*Timp1*), member RAS oncogene family (*Rab5b*), gamma synuclein (*Sncg*), and solute carrier organic anion transporter family, member 3a1 (*Slco3a1*) had high expression in DRG neurons compared to CGNs; and CAS1 domain containing 1 (*Casd1*) had high expression in CGNs compared to DRG neurons (Table S1). To characterize the correlation between FPKM and qPCR (Figure 3A; Table 2), a Kendall's correlation coefficient was computed, which ranges from -1 to +1 like the usual Pearson correlation coefficient, but makes minimal distributional assumptions. The correlation coefficient is 0.525 which is statistically significant (p = 0.0024). The probability of concordance computed from tau is 0.763. Concordance between two paired observations, (FPKM _1_, qPCR_ 1_) and (FPKM _2_, qPCR_ 2_), occurs when FPKM _1_- FPKM _2_ and qPCR_ 1_- qPCR_ 2_ have the same sign, where the subscript indexes the observation. Under the null, the probability of concordance is 0.50. All of this data supports the idea that RNA-Seq faithfully represents transcript expression [21].

**Table 3:** Genes and isoforms analyzed by qPCR.

	FPKM	QPCR
TIMP1 CGN/DRG	0.008111	0.013377
CASD1 CGN/DRG	1.546914	1.999634
RAB5B CGN/DRG	0.305764	0.442207
SLCO3A1 CGN/DRG	0.319529	0.088551
SNCG CGN/DRG	0.000423	0
APLP1 CGN/DRG	0.866076	0.866076
ATF3 CGN/DRG	0.048668	0.032352
ATF3 J1 CGN/ATF3 CGN	1.976821	0.146519
ATF3 J2 CGN/ATF3 CGN	1.239579	0.611443
ATF3 J3 CGN/ATF3 CGN	0.853864	0.844696
APLP1 CGN/ATP1A3 DRG	0.866076	0.860883
MATN2 J1 CGN/MATN2 CGN	0.846136	3.007829
PTEN J2 CGN/PTEN CGN	0.133626	0.139335
ATF3 J1 DRG/ATF3 DRG	0.227722	0.03412
ATF3 J2 DRG/ATF3 DRG	1.418459	0.098662
ATF3 J3 DRG/ATF3 DRG	0.1638	0.167082
PTEN J2 DRG/PTEN DRG	0.245028	0.002787
MATN2 J1 DRG/MATN2 DRG	2.453454	1.490104

The number in the FPKM column reflects the fold change for the given comparison. The number in the QPCR column reflects the relative expression determined using the delta, delta Ct method.

**Table 4:** Multiple transcripts were assembled by Cufflinks for many genes with known roles in axonal regeneration.

	Number of:				
Official Gene Symbol	Isoforms	TSS	CDS	3′UTR	Reference
Adcyap1 (Pacap)	2	2	2	2	Neuroscience **151**:63–73
Atf3	4	4	2	3	J Neurosci **27**:7911–7920
Bex1	2	2	2	2	J Neurochem **115**:910–920
Gap43	1	1	1	1	Development **128**:1175–82
IL-6	1	1	1	1	J Neurosci **24**:4432–43; J Biol Chem **283**:416–26
Il6st (Gp130)	1	1	1	1	Neuron **64**:617–623
Jun	1	1	1	1	Neuron **43**:57–67
Klf4	1	1	1	1	Science **326**:298–301
Klf6	1	1	1	1	Science **326**:298–301
Klf7	2	2	2	2	Science **326**:298–301
Lif	1	1	1	1	J Neurosci **21**:7161–70
Mapk8ip1 (JIP1)	3	3	2	1	J Neurosci **30**:7804–7816.
Matn2	1	1	1	1	J Cell Sci **122**:995–1004
Mdk	1	1	1	1	J Neurosci Res **87**:2908–2915.
Mtap1b	1	1	1	1	J Neurosci **24**:7204–7213
Nosip	2	2	2	2	J Neuropathol Exp Neurol **60**:411–21
Npr2	3	3	3	3	J Neurosci Res **86**:3163–9
Ptprs (PtpSigma)	4	4	4	3	J Neurosci **22**:5481–91; Science 326:592–596
Pten	3	3	3	3	Science **322**:963–966; J Neurosci **30**:9306–15
RhoA	1	1	1	1	J.Neurosci **29**:15266–76
Rock2	3	3	3	3	J.Neurosci **29**:15266–76
Smad1	2	2	1	1	J Neurosci **29**:7116–23
Socs3	1	1	1	1	Neuron **64**:617–623
Stat3	2	1	2	1	J Neurosci **26**:9512–9
Stk25 (Mst3b)	2	2	2	2	Nat Neurosci **12**:1407–14
Tnfrsf19 (TROY)	1	1	1	1	Neuron **45**:353–359
Trpc4ap	1	1	1	1	J Biol Chem **283**:416–426

The total number of transcripts expressed from each gene is in the isoform column. The total number of isoforms with: different transcription start sites (TSS), coding DNA sequences (CDS), and 3′ untranslated regions (3′ UTR) is listed below the column header. The paper demonstrating a role for the gene in axonal regeneration is listed in the Reference column.

Three of these genes, *Atf3, Pten*, and matrilin2 (*Matn2*) are regeneration-related genes [22]–[24] that generate a total of 8 isoforms. A major goal of our research is to understand how differences in gene expression confer differences in neuronal cell type specific function; in particular, to unravel the gene expression programs underlying axonal regeneration. Since DRG neurons are known for their ability to rapidly regenerate axons [9], [10], [25], further validation studies were performed on genes involved in this process.

*Atf3* is involved in peripheral nerve regeneration [22], [26]. Four *Atf3*isoforms were identified in our analysis (Figure 3B). These four isoforms differ in TSS, and one differs in the CDS (Figure S1). To validate their existence primers were designed to detect differences in the TSS and to identify the predicted change in CDS in isoform Atf3 J3. *Atf3, Atf3 J1* and *Atf3 J2* were amplified and validated by sequencing (Figure 3B and data not shown). Although we amplified an *Atf3 J3* isoform containing the 4^th^ TSS, we were unable to identify an *Atf3 J3* sequence containing the predicted change in CDS. Thus the four predicted TSS's were validated but the predicted change in CDS could not be confirmed using this PCR based strategy.

Another gene of interest in neuronal regeneration is *Pten*; conditional ablation of *Pten* results in impressive axonal regeneration in retinal ganglion, corticospinal tract, and DRG neurons [23], [27], [28]. We identified 3 Pten isoforms in our analysis (*Pten, Pten* J1, and *Pten* J2; Figure 3C). *Pten* J1 is identical in sequence to the conventional *Pten* isoform except for a difference in TSS and a small shift in splice site around exon 5 and 6 that is predicted to result in a two amino acid change. Using PCR amplification and sequencing analysis we were unable to confirm the existence of this small shift in coding sequence (data not shown). We validated the existence of *Pten* J2 through both PCR amplification and sequencing (Figure 3C). *Pten* J2 has a truncated CDS, an alternative transcription start site and a longer 3′UTR compared to the conventional *Pten* isoform expressed within neurons. *Pten* J2 expression was lower in DRG neurons and CGNs compared to the conventional *Pten* isoform, a result confirmed by qPCR analysis (Figure 3D, E). The truncated CDS encodes a protein that lacks the phosphatase domain but maintains an intact C-terminal domain (Figure S2D). Available antibodies were unable to confirm the presence of PTEN J2 protein in DRG neurons based on distinct bands on western blots (data not shown). To begin to understand PTEN J2 protein function, we expressed both conventional PTEN and PTEN J2 from cDNAs (Figure S2A–C). We hypothesized that overexpression of conventional PTEN would suppress neurite outgrowth due to negative regulation of phosphatidylinositol (3,4,5)-trisphosphate (PIP_3_) and serine/threonine protein kinase Akt, key regulators of neurite outgrowth [29] and that overexpression of PTEN J2 could potentially act as a dominant negative for PTEN due to the predicted lack of the phosphatase domain (Figure S2C–D; [30]). Protein products of the predicted size for PTEN and PTEN J2 could be detected by Western blotting after transfection of 293T cells (Figure 3F). Perhaps surprisingly neither overexpression of PTEN nor that of PTEN J2 in primary cortical neurons significantly affected neuronal morphology (Figure S2E), despite PTEN's known role in suppression of axon growth [31]. We conclude that overexpression of PTEN and PTEN J2 alone are not sufficient in primary cortical neurons to negatively regulate PIP_3_ to such a degree that Akt mediated neurite outgrowth is affected.

### Binding Site Analysis Predicts Cell Type Specific Transcriptional Networks

**Figure 4:**
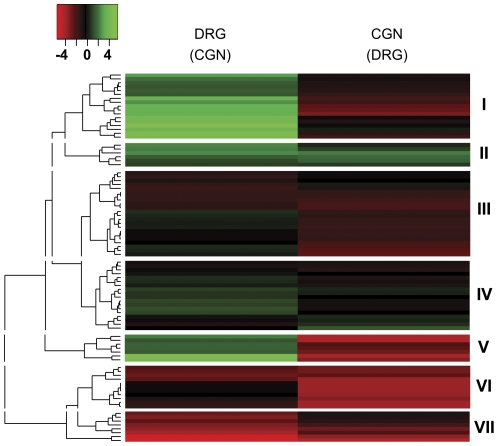
Hierarchical clustering of transcription factor binding sites in differentially expressed isoforms. Heat map showing Z-scores reflecting the frequency of binding sites for TFs found in the promoters overexpressed for each comparison. Green indicates high relative abundance for the TF binding site, and red low relative abundance. Clusters I and V represent TFBSs whose binding sites are enriched in DRGs. Cluster III, IV, VI and VII consists of TFBSs with low abundance in all comparisons.

RNA-Seq data can be used to identify precise exon locations that in turn allow the determination of TSSs for each expressed isoform. Scanning individual isoform promoters for TF binding sites allows prediction of TFs regulating specific isoforms [32]. By doing this it is possible to uncover clusters of expressed isoforms mediated by cell-specific factors [32]–[34]. Thus we identified promoter regions for each isoform found to be overexpressed in one neuronal type compared to the other, and then used ASAP, an online tool, to estimate the relative abundance of TF binding sites (TFBSs) in these two groups of promoters (compared to a “background” set of randomly chosen promoters [33]. Relative abundances were calculated for each TFBS in each of the two groups of promoters (from transcripts overexpressed in DRG neurons or overexpressed in CGNs), and these values were used to develop a hierarchical cluster of the TFBSs, in the form of a heat map (Figure 4; Table S2). At one level on the map, TFBSs grouped into 7 clusters; TFs in clusters I and V were enriched in DRG neuron-overexpressed isoforms while the majority of TFs found in clusters III, VI, and VII showed no enrichment in either group of overexpressed transcripts (Figure 4). Interestingly, most TFBSs found to be overrepresented within the promoters of differentially expressed isoforms were in the promoters of DRG neuron-selective isoforms.

### MicroRNA response element analysis of 3′UTRs

RNA-Seq identifies not only TSSs, but also the full 5′ and 3′ untranslated regions (UTRs) of transcripts. The 3′UTR is thought to be the main target region for miRNAs, which bind to mRNAs and mediate their degradation or inhibit their translation [34]. Since miRNAs play important roles in cell-type definition [35]–[37], we used an approach similar to that used for the TFBSs to predict miRNAs involved in individual isoform regulation. Using the same 2 groups of differentially expressed isoforms, we analyzed the relative abundance of miRNA Response Elements (MRE). Since miRNA binding generally leads to mRNA downregulation, a relative paucity of MREs would be expected to correlate with increased activity of the relevant miRNA on that group of transcripts.

**Figure 5:**
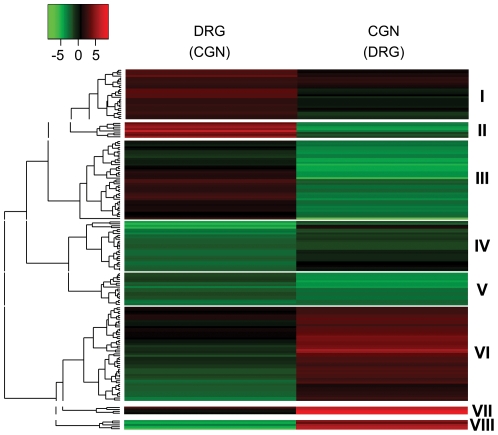
Hierarchical clustering of miRNA target sites in the 3′ UTRs of differentially expressed isoforms. Heat map representing Z-scores derived from MRE frequency. Red indicates a frequent appearance of the MRE (suggesting low miRNA activity in the corresponding cell type) and green a low frequency (suggesting possible high miRNA activity in the corresponding cell type). Cluster II contains miRNAs with responsive elements appearing in 3′UTRs of transcripts overexpressed in DRGs. Cluster I miRNAs are present in most of the transcripts regardless of comparison indicating these miRNAs may not be active in neurons. Clusters VI-VIII represent miRNAs that could be potentially active in DRG enriched transcripts due to their low appearance in DRG 3′UTRs, while Cluster II and III represent miRNAs potentially active in CGNs. Cluster IV and V represent miRNAs with a low abundance of target sites regardless of cell type suggesting these miRNAs may be active in both neuronal types.

Groups of 3′UTRs were submitted to TargetScan, which allowed us to scan for known and registered MREs [38]. Relative abundance and frequency of miRNA target sites in the groups were manually calculated following a model developed for TFBS analysis [39], which underlies ASAP, and the resulting Z-scores were used for hierarchical clustering (Figure 5; Table S3). Cluster analysis revealed groups of co-regulated miRNAs. To facilitate analysis of miRNAs we chose a level of the dendogram that defines 8 clusters. Clusters II and III contained miRNAs whose target sites were found in low abundance in CGN-enriched 3′UTRs suggesting high miRNA activity (Figure 5). In contrast, the miRNAs in cluster VI, VII, and VIII were found in low abundance in DRG 3′UTRs, suggesting high activity of the cognate miRNAs. Further validation of these miRNAs in these cell types is needed to draw conclusions about cell-type specific regulation, but the strong differences in MRE abundance suggests that this approach can lead to the identification of relevant miRNA targets, and that these may help shape neuron specific isoform expression.

### Using TFBS and miRNA data for network analysis

**Figure 6:**
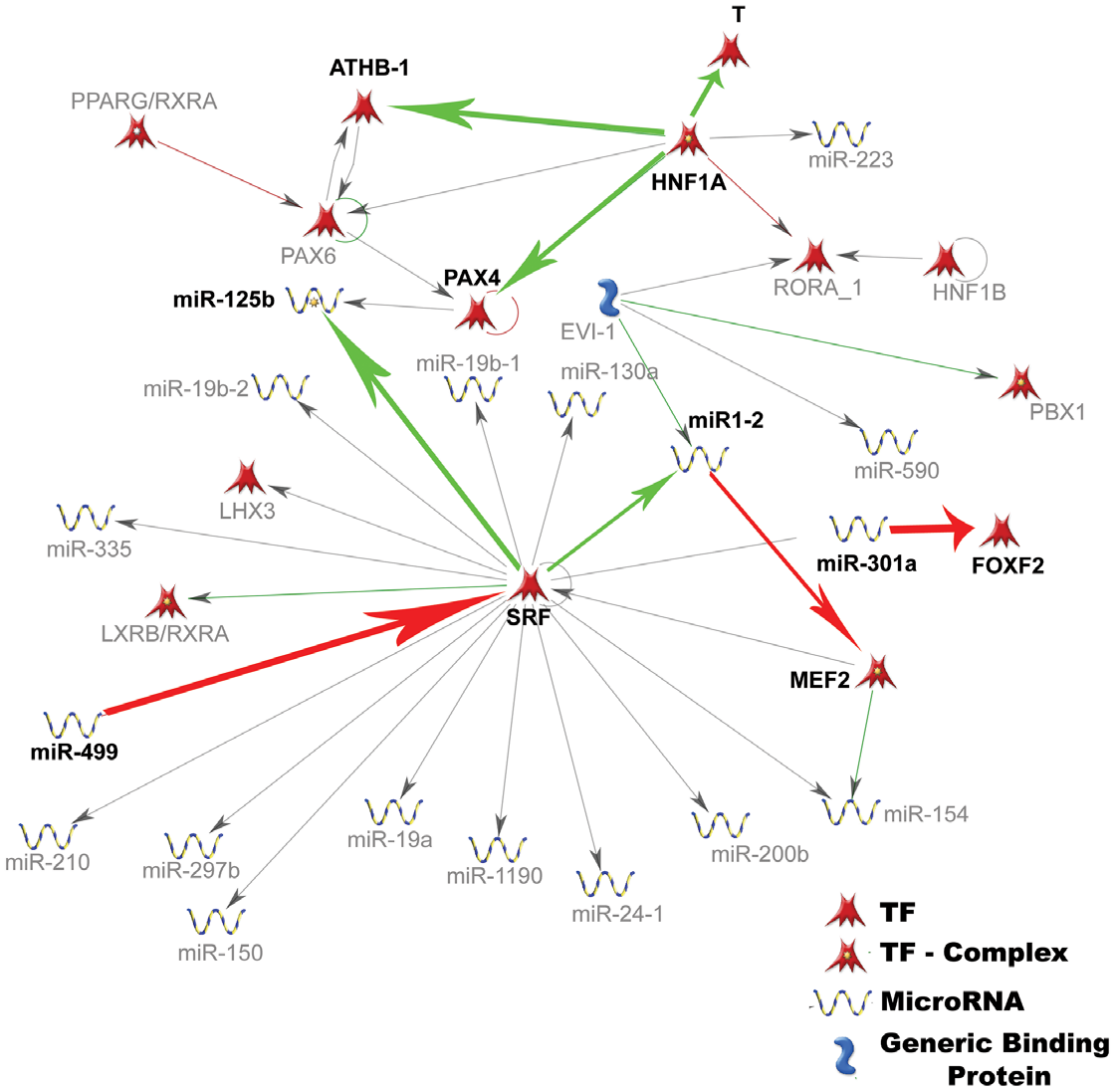
Interaction networks between TFs and miRNAs. The shortest paths algorithm was used to examine interactions between the TFs in Clusters I and V (Figure 4) and the miRNAs in Clusters II, VI, VII, and VIII (Figure 5; GeneGo, MetaCore, Inc,). The number of steps was limited to one. All interactions are shown. Interactions that support the findings from TFBS and miRNA clustering are indicated in bold. Green indicates activation and red inhibition.

Understanding the functional nature of differential gene expression has traditionally involved the use of Gene Ontology but this method considers genes and is not yet implemented for isoforms. Therefore our approach was to identify the regulatory events directing isoform diversity. We did this by examining the TFBSs and MREs on differentially expressed isoforms. By crossing the TFBS data with the miRNA analysis we predicted novel interaction networks potentially active in these neuronal types. GeneGo MetaCore was used to generate an interaction network between clusters I and V from the TFBS analysis (Figure 4), and clusters II, III, VI, VII, and VIII from the miRNA analysis (Figure 5). The network suggests that miR-499 negatively regulates serum response factor (*SRF;* Figure 6; [40]). While this is a known interaction, it is supported by the predicted activity of both miR-499 and SRF in DRG neurons: miR-499 target site was found in high abundance in DRG neuron 3′UTRs, suggesting low activity and SRF TFBSs are found in high abundance, suggesting high SRF activity. Another potential miRNA-TF interaction was identified between miR-125b and SRF (Figure 6; [41], an observation which is yet again consistent with the predicted activity of these two molecules; Figure 4 and 5). These two examples demonstrate that this approach, to analyze differentially expressed isoforms for TFBS and miRNA target sites, can associate specific TFs and miRNAs activity previously identified in other systems to neuronal functions or identity. We posit that the SRF may be a key transcriptional regulator to promote axon growth. It is known that SRF mediates NGF dependent axon growth and DRG neuron target innervation in early development [42] and here we show that it has numerous predicted interactions with miRNAs (Figure 6). This makes *Srf* a prime candidate for activation because it has the potential to regulate numerous genes simply by its ability to impact the expression of multiple miRNAs in a cell type (DRG neurons) exhibiting robust neurite growth both in vitro and in vivo after an axonal injury. Indeed while there is no change in the expression of serum response factor (*Srf*), we find that a novel isoform of serum response factor binding protein (*Srfbp1*) is significantly overexpressed (up to 10-fold higher) in DRG neurons (Table S1). The activity of the other TFs and miRNAs can be tested in future experiments for their relevance in the specific cellular populations.

## Discussion

In our studies we applied RNA-Seq to two neuronal populations: cerebellar granular neurons (CGNs) and dorsal root ganglion (DRG) neurons. While a few recent studies have utilized RNA-Seq on nervous tissue [7], [8], RNA-Seq on distinct neural types remains largely unexplored [43]. To acquire pure cellular populations cells were cultured in conditions such that large numbers of nearly pure CGNs [12] and DRG neurons were obtained (see Materials and Methods). We were able to identify differentially expressed isoforms (Figure 2), confirm the identities of numerous novel isoforms and validate their expression levels (Figure 3), and perform a bioinformatic analysis to understand isoform expression regulation (Figure 4, 5, 6).

Why is understanding isoform diversity important? It is well accepted that genes express multiple isoforms and recently it has been demonstrated that isoform number increases with sequencing depth [44]. Identifying all expressed isoforms in specific cell populations is therefore necessary to fully understand all of the components contributing to cellular function. In addition to the sheer number of isoforms with unknown functions, numerous studies prove that isoforms can be functionally different [5], [6]. In our dataset two genes of interest, *Atf3* and *Pten* produced multiple isoforms (Figure 3). We used PCR and sequencing to confirm 3 TSSs for *Atf3* (Figure S1). One alternative transcription start site in our data was previously identified and characterized [45], but never annotated; the fact that we found this TSS demonstrates the reliability of this approach. Each *Atf3* promoter is active under different conditions. *Atf3 J1* and *J3*, the two isoforms with the lowest expression in DRG neurons, use the P1 promoter which is primarily active in response to stress and in numerous cancers [45], whereas the conventional *Atf3* promoter (P2) typically is reactive to mitogenic stimuli [46]. The *Atf3* isoform with the highest FPKM in DRG neurons was J2 and its promoter is completely novel (Figure S1). While the CDS for these isoforms are the same, one way to confer functional differences could be through differential promoter regulation which could cause functional differences simply by changing coregulated genes.

Another point of isoform diversity occurs with alterations in the CDS and 3′UTRs. Changes in CDS may lead to the most obvious alterations in function as whole protein domains with specific functions could be present or absent. We examined numerous other transcripts associated with neuronal regeneration and found that many harbored changes that would affect the CDS (Table 4). One such validated example is the discovery of *PTEN J2*. *PTEN J2* uses an alternative TSS and has a longer 3′ UTR compared to the *PTEN* transcript expressed in these neuronal cells (Figure 3B). The predicted open reading frame indicates that protein made from this transcript would lack the phosphatase domain (Figure S2). We were not able to validate the presence of endogenous *PTEN J2* using existing antibodies. This could be because endogenous protein expression is low (consistent with the low expression of the J2 transcript), but it is also possible that *PTEN J2* exists solely as a regulatory noncoding RNA (ncRNA), which could potentially function as a miRNA sponge to participate in the fine regulation of the main transcript [47]–[49].

Why analyze differentially expressed isoforms for specific TFBSs and for miRNA response elements? If one hopes to truly understand the regulation driving isoform diversity, it is necessary to identify the transcription factors and miRNAs that direct their expression. The idea that this approach identifies novel regulatory networks is supported by a comparison between the present study and a previous study performed in our lab. In that study, TFBS analysis was performed on differentially enriched genes between DRG neurons and CGNs after subtractive hybridization and microarray analysis [50]. Interestingly, we found only 6 TFs that overlap between the two studies (*Cepba*, *Irf1*, *Myc*, *Pax4*, *Rel*, and *Tead*). Some differences may be attributed to the TFBS matrices used. Smith et al. used TRANSFAC to examine specific binding sites, while in this study we employed the JASPAR database, which examines TFBS matrices. In addition, different cutoffs for differential gene expression between the two studies likely account for some of the differences observed. It will be interesting to test whether the 6 TFs common to both analyses may be network hubs driving DRG neuron specific gene expression.

In addition, network analysis could identify novel and functionally relevant pathways. In fact, we found that numerous interactions first identified in muscle tissue [40], [41], were predicted to be active in DRG neurons. For instance, our TFBS analysis predicted high activity of SRF, forkhead box F2 (FOXF2) and myocyte enhancer factor 2 (MEF2) in DRG neurons, which is consistent with the high abundance of response elements for miRNAs-1-2, -301 and -499 in DRG neuron-enriched transcripts (Figure 6; [40], [51], [52]). Previous identification of these interactions in muscle cells supports the existence of this network. When we examine the expression of SRF, MEF2A, and FOXF2 in our dataset, we find that SRFBP1 and a novel isoform of MEF2B are significantly overexpressed in DRG neurons compared to CGNs (Table S1), supporting the idea that they may be more active in DRG neurons. Overall, this study profiles the isoform diversity found in two neuronal populations, reports differential isoform expression, and identifies potential regulatory networks active in each population. We conclude that applying RNA-Seq to distinct neuronal populations can uncover the rich isoform diversity that contributes to neuronal identity and differential function.

### Ethics Statement

All procedures using animals were approved by the University of Miami Animal Care and Use Committee.

## Materials and Methods

### DRG and CGN Cell Culture

DRGs and CGNs were cultured from P8 C57bl/6j mice. CGNs were isolated as previously described by our laboratory [53]. Tissue culture plate preparation was performed as previously described [54]. From one mouse, 5×10^5^ cells were grown for 16hrs on tissue culture dishes coated with 100ug/mL poly-d-lysine (Sigma-Aldrich) and 5ug/mL laminin (Trevigen, Inc.). Two biological replicates for each CGN substrate were prepared for RNA-Seq. For qPCR validation, three additional biological replicates were prepared exactly as for the RNA-Seq experiment.

For DRG isolation, each ganglion was trimmed of its axons, and then incubated in a solution of dispase (10mg/mL; Invitrogen), trypsin (0.25%; Invitrogen), and collagenase (3000U/mL; Invitrogen). Fetal bovine serum was used to quench the trypsin. Cells were centrifuged at 80G and then resuspended in L-15 media (Invitrogen) and DNase (0.2mg/mL; Sigma Aldrich) followed by trituration. Ganglia from two mice were combined for each biological replicate. Three biological replicates were prepared in total. The DRG media was prepared as previously described [50] and supplemented with 0.05 µg/mL nerve growth factor and 10 µM 5′-fluoro-2′deoxyuridine (FuDR, Sigma) to eliminate contaminating glial cells. DRG cultures underwent three rounds of 4-day FuDR treatment over the course of 16 days. Cell culture purity was determined by counting the number of neuronal specific tubulin positive cells and comparing that to number to Hoechst positive nuclei using the Cellomics ArrayScan VTI automated imaging system (Thermo Scientific). Cultures were 74% neuronal and examination of FPKMs for some known glial associated genes supports that finding. The FPKMs for the two known isoforms of glial fibrillary acidic protein were 11 (uc007lsw.1) and 32 (uc007lsx.1). The FPKM for myelin protein zero (Mpz) isoforms was 11,252 FPKM and for myelin protein like zero 1 and 3 the FPKMs were 115 (Mpzl1-uc007djg.1), 634 (Mpzl1-uc007djh.1), 769 (Mpzl1-uc007dji.1), 4 (Mpzl3-uc009pfb.1), and 157 (Mpzl3-uc009pfc.1). The FPKMs for myelin basic proteins were 2979 (Mbp- uc008ftx.1), 5471 (Mbp- uc008ftw.1), and 10831 (Mbp- uc008ftz.1). While the FPKMs for Mpz and Mbp appear high it is worth noting that DRG neurons have been demonstrated to express both transcripts (Allen Institute for Brain Science and Eurexpress).

### RNA Isolation & Preparation for Next Generation Sequencing

RNA was extracted using Trizol Reagent (Invitrogen) following standard methodology. The RNA Integrity Number (RIN) was greater than 9.5 for each biological replicate. Next Generation Sequencing was performed at the Hussman Institute for Human Genomics Sequencing Core Facility (University of Miami, Miami, FL). RNA was prepared for Next Generation Sequencing following the Illumina mRNA Sample Preparation Guidelines (Illumina, Cat # RS-930-1001). Each sample was run over two lanes and subjected to 52 sequencing cycles on the Illumina Genome Analyzer II (Illumina). Image analysis and base calling were performed using Genome Analyzer II Pipeline v1.5 (Illumina). Read alignment using the Efficient Large-Scale Alignment of Nucleotide Databases (ELAND) algorithm is part of the Illumina pipeline and standard service at the sequencing core. 80% or more of the reads aligned to the mouse reference genome (mm9) which is similar to what was found in our analysis using Bowtie [15].

### Bioinformatics

All of the bioinformatic analyses were run on the “Pegasus”, a Linux based supercomputer with 5000 central processing units (http://www6.miami.edu/miami-magazine/featurestory2.html).

#### Reads alignment with Tophat and Bowtie

Raw reads were first aligned to the mouse reference genome (assembly mm9). For this purpose we used the Tophat software, version 1.0.13 [14], [15]. Default settings were used except for the following options: –G option which supplies Tophat with gene model annotation (combined UCSC, Ensembl, and RefSeq annotations) and –i 50 which sets the minimum intron length to 50. The software works through the Bowtie fast aligner and it is able to identify reads that entirely map to the reference genome as well as predicting splice junctions aligning reads that span across distant areas of the genome without any reference annotation. This process was performed independently for each single sample. On average 79% of reads aligned in at least one region of the reference genome for all the samples (Table 1).

#### Transcript reconstruction and expression estimation

Aligned reads were assembled into the different RNA-species by the Cufflinks software (version 0.8.3). At first Cufflinks uses the aligned reads in the dataset to describe a set of transcripts starting from the reads that span splice-junctions. We ran this step using a non-annotated reference genome because without an annotation the software will assemble novel transcripts and isoforms. After transcript assembly, normalized expression levels are estimated and reported as FPKM (**F**ragments **P**er **K**ilobase of exon per **M**illion fragments mapped) together with confidence intervals. A different part of the software, named Cuffcompare (version 0.8.3) classified the reconstructed RNA-species as novel or known according to how they map back to the provided reference annotation [16]. Cuffcompare was run twice, first with a combined reference GTF generated from crossing annotated transcripts found in the UCSC Genome, the Ensembl, and the RefSeq database. We combined three genome annotations in an effort to minimize falsely identified novel transcripts. UCSC was used as the base since it contained the highest number of annotated RNA species; all non overlapping annotations found in the other databases were added. We reran Cuffcompare in order to improve the accuracy of read alignment and therefore transcript expression estimation (personal communications with Cole Trapnell). The nature of the alignment of the reconstructed RNA-specie and the annotated element are reported according to a code letter. For our purposes we isolated from the output only the “ = ”, “j” and “u” classes (corresponding to a “perfect match” to a known RNA-molecule, new isoforms of known active locus, and to full transcripts never identified before – see [16] for more details). We ran Cuffcompare a 2nd time after adding annotation for unknown and novel transcripts assembled by Cufflinks.

#### Differential Expression Testing

In order to determine which isoforms were differentially expressed within the dataset we used Cuffdiff [16]. Cuffdiff allowed the biological replicate data to be run by Cufflinks as a group, thus enabling identification of differentially expressed isoforms between conditions. Isoforms were considered significant if they met Cuffdiff's requirements to perform a statistical test (see Materials and Methods; [16]), had a corrected p-value <0.05, and an absolute value of the natural log of the fold change >1. Cuffdiff (version 0.9.3) was run using the new combined annotation. We used the upper-quartile normalization option to exclude reads coming from highly expressed genes which allowed more accurate expression level determination of transcripts expressed at low levels [55]. Cuffdiff was run twice: the first time expression estimation was performed separately for each single sample, allowing us to assess the variability between biological replicates. The second run was performed using the “biological replicates” option. This option gave a single expression level per transcript per condition and allowed differential expression testing between cell types. Cuffdiff determined statistical significance based on the square root of the Jensen-Shannon divergence between the relative abundance of transcripts [16]. Significance was reported as an uncorrected p-value, and then classified as significant/not –significant after Benjamini-Hochberg correction of the p-value. We considered transcripts as differentially expressed if: 1) the Jensen-Shannon test statistic was reported, 2) the False-Discovery-Rate adjusted p value was less than 0.05, and 3) change in relative abundance in either direction was e-fold or greater, e is the base of the natural logarithm.

### Logistic Regression Analysis

Based on the 95% confidence intervals for FPKM produced by Cufflinks software, we distinguished more reliable from less reliable FPKM estimates by labeling an FPKM estimate with a lower confidence bound exceeding 0 as “reliable” and an FPKM estimate with lower confidence bound of 0 as “unreliable”. We used these observations to fit a logistic regression function (SAS, version 9.2) relating the probability that an isoform was reliable to log FPKM.

### Defining TSS, CDS, and 3′ UTR

TSSs were defined as the beginning of the first exon. A change in CDS was defined as any change that occurred from exon 2 to the second to last exon when compared to the conventional isoform. 3′ UTRs were defined as the last exon. The number of differentially expressed isoforms associated with a CDS in the reference annotation was greater than 93% indicating that the vast majority of transcripts analyzed in this dataset are in fact messenger RNAs and not noncoding RNAs.

### Quantitative Real Time PCR

Three additional biological replicates were created for both DRGs and CGNs. RNA from these replicates was used as input (450ng) for a reverse transcription reaction using oligo d(T) primers following the manufacturer's recommendations (Advantage RT-for PCR kit, Clontech). In all cases primers designed for qPCR spanned exon-exon boundaries. Real time PCR was performed using 2X SYBR Green master mix (Applied BioSystems) on the Gene Rotor System (Corbett Research, Qiagen). Relative expression was calculated using the delta delta C_t_ method [56]. Primer sequences can be found in Table 5.

**Table 5:** Primer sequences used for qPCR and isoform validation.

Gene/Isoform	Primer Sequence	Product Size (bp)
Atp1a3	5′-CCCCATATCTTCTTTAGGGTCTG-3′	144
	5′-GCAGGATAGAGAAGCCACCA-3′	
Aplp1	5′-CCTTCAGGTGATCGAAGAGC-3′	124
	5′-ACTGGGACCCAAGTGTTCAG-3′	
Atf3	5′-CCAGCCACAGTCTCACTCAG-3′	1435
	5′-CAACAGAGGATGGACGACAC-3′	
Atf3 J1	5′-TGGAAGAGAGACTCCTCTGAACA-3′	1339
	5′-CAACAGAGGATGGACGACAC-3′	
Atf3 J2	5′-AGATCCAATCCCTGCCTTG-3′	1289
	5′-CAACAGAGGATGGACGACAC-3′	
Atf3 J3	5′-CAGACCAGACAAGAGTATGGAAGA-3′	1072
	5′-TTTCCGGGAGTTTCATCAGA-3′	
Timp1	5′-ATTCAAGGCTGTGGGAAATG-3′	183
	5′-CTCAGAGTACGCCAGGGAAC-3′	
Sncg	5′-GACCAAGGAGGGGGTTATGT-3′	135
	5′-ACTGTGTTGACGCTGCTGAC-3′	
Slo3a1	5′-TCTTATGCGCTGGGAGTTCT-3′	106
	5′TGCTCCAGAACAGACAGGTG-3′	
Casd1	5′-AGCAGCACCAGGACCTCTAA-3′	114
	5′TCTGCTCGATTCAGGAAGGT-3′	
Rab5b	5′-GAAGTTGCCAAAGAGCGAAC-3′	220
	5′-CAGGGCTCAGTGTGCTGTTA-3′	
Matn2 J1	5′-CCTGAGCCAGTCACCATAAA-3′	278
	5′-TTTAGGCGATTTTCCAAAGC-3′	
Matn2	5′-AGCCAACAGTGCAACATAGA-3′	161
	5′-TTCATTTGCAACGTTCTGGA-3′	
Pten	5′-GGATTTCCTGCAGAAAGACTTG-3′	187
	5′-GCTGTGGTGGGTTATGGTCT-3′	
Pten J2	5′-CACTGGCTCCAGATTGTAGG-3′	244
	5′CGTCCCTTTCCAGCTTTACA-3′	
Smad1	5′-CAGCGCGACCAGATCAAT-3′	746
	5′-AGTGGTAGGGGTTGATGCAG-3′	
Smad1 J1	5′-TTTGTTTCTGCCCTGAGCTT-3′	591
	5′-AGTGGTAGGGGTTGATGCAG-3′	

### Validation of Atf3 and Pten Isoforms

Sequences for each set of isoforms were retrieved from the UCSC Genome Browser after uploading a user supplied GTF with the coordinate locations of each exon in the *Atf3* and *Pten* isoforms. Primers for validation can be found in Table 5. PCR results in Figure 3B-C were obtained using DRG cDNA, with 4.5ng of DRG RNA (no RT-control), or with water (no template control).

### Pten isoform over expression

*Pten* and *Pten* J2 CDS's were cloned into a modified pAAV-MCS plasmid (University of Miami Viral Vector Core, Miami, FL) containing the 2A peptide (Figure S1). The 2A peptide facilitates a ribosomal pause and thus produces two individual proteins from a polycistronic mRNA [57]. Pten-2A-eGFP/mCherry or Pten-J2-2A-eGFP/mCherry were transfected into HEK293 cells (Fugene, Roche) followed by Western blotting for PTEN or PTEN J2 (Cell Signaling, #9559). Early postnatal cortical neurons were prepared and transfected as previously described [58]. All plasmids (*DCX*, *ORX1*, *PTEN*, *PTEN* J2) were overexpressed from the pAAV-2A plasmids. Transfected cortical neurons were grown for 3 days, fixed and immunostained with Hoechst dye to mark nuclei and for neuronal specific tubulin (mouse monoclonal antibody, produced at the University of Miami monoclonal antibody core facility). Neurons were grown at densities such that processes could be accurately imaged and traced without substantial overlap between cells. Neuronal imaging and tracing was performed using the Cellomics ArrayScan VTI automated microscope (Thermo Fisher). Neurite length was determined using the Neuronal Profiling Algorithm v3.5. Data analysis was performed in Spotfire Decision Site Software (v9.1.2, Tibco). Transfected neurons were determined by calculating a background fluorescent intensity in neurons that did not receive plasmid and then a threshold was set, above which a neuron was deemed transfected [58]. The total neurite length for transfected neurons was determined for each plasmid condition. Dunnett's post test was performed to determine if there were significant differences in neurite length after transfection. OXR1 served as the neutral control and DCX as a positive control [58].

### Transcription Factor Binding Sites Analysis

Differentially expressed transcripts were grouped according to their expression profiles. Promoter regions -1000 to + 300 bp from the TSS were isolated for each single transcript. We took advantage of individual start sites as identified by sequencing to define RNA-species specific promoters.

Matrix models for Vertebrate Transcription Factors Binding Sites (TFBS) from the JASPAR database were then used to scan the promoter regions [59]. The frequencies of the binding sites were calculated as number of binding sites per base pair independently for each group and then compared to the frequencies calculated in a background of 5000 randomly chosen promoters to obtain the expected frequencies. The whole analysis was performed by Asap [33], which reported over or underrepresentation in each group as a Z-score calculated on the basis of frequency of TFBS appearance. We used only transcription factors with a Z-score one standard deviation above and below the average Z-score in at least one of the conditions to draw the heatmap.

### MicroRNAs Target Site Analysis

The same groups of transcripts used for the TFBS analysis were tested for abundance of target sites for specific miRNAs. MiRNAs are thought to target primarily 3′UTR's of messenger RNAs although recent reports have demonstrated that functional target sites can be found even in the coding sequence as well as in the 5′UTR of the transcripts [60], [61]. We decided to test the 3′UTR as the originally identified location of miRNA targets and therefore the one with the better characterized binding nature. Considering the complexity of defining the exact coding sequences and demonstrating their functionality, we defined 3′UTRs as the last exon of each transcript. MiRNA target sequences were based on the 5.1 release of TargetScanMouse (http://www.targetscan.org/mmu_50/; [38]). We allowed the software to recognize only perfect match complementarities to the seed-region of each miRNA. Three different kinds of sites are then reported: 7mer-8m, 7mer-1A and 8mer (see [38]).

To test for significance of over or underrepresentation of target sites in the different groups the approach described for the TFBS analysis was used. Briefly, frequencies of target sites per base pair were calculated for each group as well as for a background of 5000 randomly chosen 3′UTRs. Frequency in each group was compared to the frequency in the background to calculate the expected frequency. Observed and expected frequencies were then used to calculate a Z-score representing over or underrepresentation of target sites in each individual group. This approach was previously described by Sui and colleagues [62]. We used only miRNAs with a Z-score +/− 1 standard deviation around the average Z-score in at least one of the conditions to draw the heatmap.

## Supporting Information

Figure S1 **Atf3 transcripts in the UCSC Genome Browser.** Labels are directly above each track display. Thick black bars are exons and the thin line indicates introns. Arrows indicate direction of transcription (*Atf3* is on the minus strand). *Atf3*, *Atf3 J1*, and *Atf3 J2* have all been reported before. *Atf3 J2* is a novel isoform. In total three promoters are active: P1, P2 and a novel promoter, P3.
(TIF)

Figure S2 **Pten isoform analysis.** *A*, *B*, pAAV-2A-Pten or -Pten J2 over expression plasmids. *C*, The 2A peptide bridge sequence which mediates a ribosomal pause resulting in two independent proteins (2A bridge schematic adapted from Tang et al., 2009). *D*, Pten and Pten J2 amino acid sequences were put into protein BLAST. Pten J2 predicted protein lacks the phosphatase domain. *E*, Total neurite length is not changed compared to control (Oxr1). Doublecortin (DCX) is a positive control as it is known to increase neurite length. ***P<0.005.
(TIF)

Table S1 **Isoforms tracking file.** The nearest reference ID column refers to associated transcript ID given by UCSC Known Genes, RefSeq, or Ensemble database. A _j indicates a novel isoform. FPKM estimation for each individual sample with confidence intervals is shown. The biological replicates FPKM information was generated in the Cuffdiff run and used to differential expression analysis.
(XLSX)

Table S2 **Transcription factor names and Z-scores that went into creating the** **Figure 4** **heatmap.** The transcription factors are from the JASPAR database (release 10/2009). The Z-score for each TFBS in the promoters of DRG or CGN enriched isoforms is shown.
(XLSX)

Table S3 **MiRNA names and Z-scores that went into creating the** **Figure 5** **heatmap.** The Z-score for each TFBS in the 3′ UTRs of DRG or CGN enriched isoforms is shown.
(XLSX)
