# Supplementary figures and images for: Isoform Diversity and Regulation in Peripheral and Central Neurons Revealed through RNA-Seq
Source: PLoS One. 2012 Jan 17;7(1):e30417. doi: 10.1371/journal.pone.0030417 (PMC3260295; doi:10.1371/journal.pone.0030417)

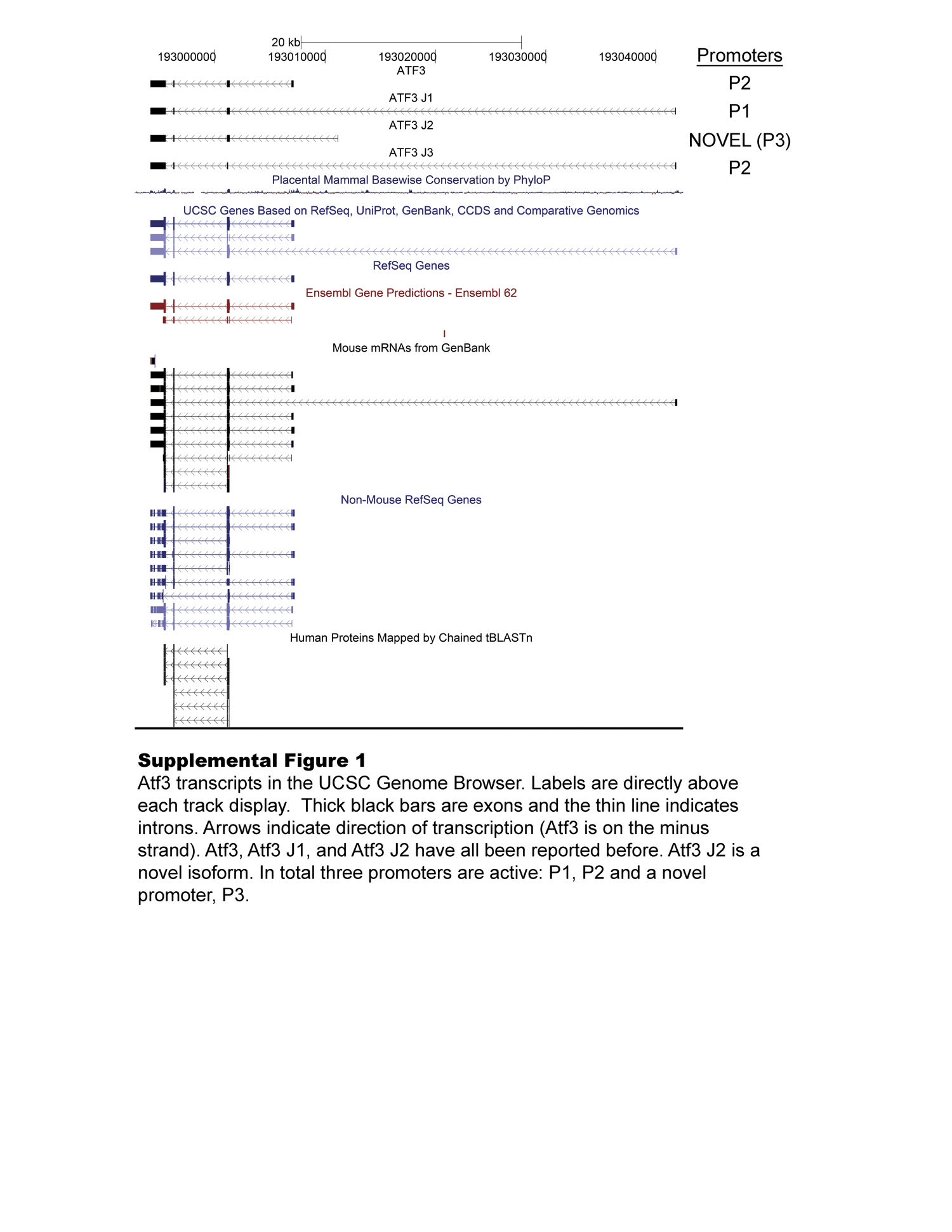

Supplement: Figure S1 — Atf3 transcripts in the UCSC Genome Browser. Labels are directly above each track display. Thick black bars are exons and the thin line indicates introns. Arrows indicate direction of transcription (Atf3 is on the minus strand). Atf3, Atf3 J1, and Atf3 J2 have all been reported before. Atf3 J2 is a novel isoform. In total three promoters are active: P1, P2 and a novel promoter, P3. (TIF) [file pone.0030417.s001.tif]

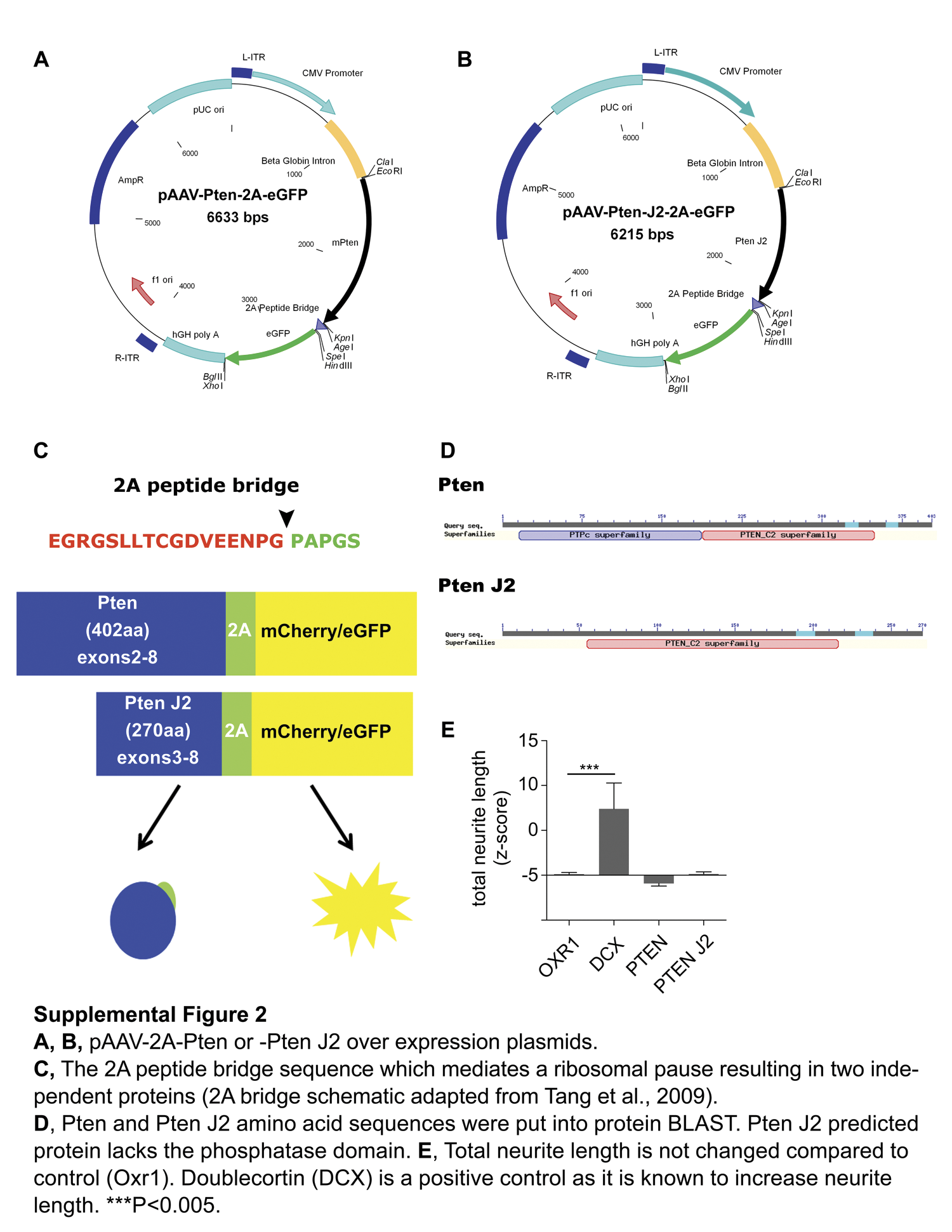

Supplement: Figure S2 — Pten isoform analysis. A, B, pAAV-2A-Pten or -Pten J2 over expression plasmids. C, The 2A peptide bridge sequence which mediates a ribosomal pause resulting in two independent proteins (2A bridge schematic adapted from Tang et al., 2009). D, Pten and Pten J2 amino acid sequences were put into protein BLAST. Pten J2 predicted protein lacks the phosphatase domain. E, Total neurite length is not changed compared to control (Oxr1). Doublecortin (DCX) is a positive control as it is known to increase neurite length. ***P<0.005. (TIF) [file pone.0030417.s002.tif]
